# The cervix as a natural tamponade in postpartum hemorrhage caused by placenta previa and placenta previa accreta: a prospective study

**DOI:** 10.1186/s12884-015-0731-9

**Published:** 2015-11-11

**Authors:** Saad A. A. El Gelany, Ahmed R. Abdelraheim, Mo’men M. Mohammed, Mohammed T. Gad El-Rab, Ayman M. Yousef, Emad M. Ibrahim, Eissa M. Khalifa

**Affiliations:** Minia Maternity & Children University Hospital, Department of Obstetrics & Gynecology, Faculty of Medicine, Minia University, Minia, Egypt

**Keywords:** Placenta previa, Placenta accreta, Postpartum hemorrhage, Cervical inversion, Tamponade

## Abstract

**Background:**

Placenta previa and placenta accreta carry significant maternal and fetal morbidity and mortality. Several techniques have been described in the literature for controlling massive bleeding associated with placenta previa cesarean sections. The objective of this study was to evaluate the efficacy and safety of the use of the cervix as a natural tamponade in controlling postpartum hemorrhage caused by placenta previa and placenta previa accreta.

**Methods:**

This prospective study was conducted on 40 pregnant women admitted to our hospital between June 2012 and November 2014. All participating women had one or more previous cesarean deliveries and were diagnosed with placenta previa and/or placenta previa accreta.

Significant bleeding from the placental bed during cesarean section was managed by inverting the cervix into the uterine cavity and suturing the anterior and/or the posterior cervical lips into the anterior and/or posterior walls of the lower uterine segment.

**Results:**

The technique of cervical inversion described above was successful in stopping the bleeding in 38 out of 40 patients; yielding a success rate of 95 %. We resorted to hysterectomy in only two cases (5 %). The mean intra-operative blood loss was 1572.5 mL, and the mean number of blood units transfused was 3.1. The mean time needed to perform the technique was 5.4 ± 0.6 min. The complications encountered were as follows: bladder injury in the two patients who underwent hysterectomy and wound infection in one patient. Postoperative fever that responded to antibiotics occurred in 1 patient. The mean duration of the postoperative hospital stay was 3.5 days

**Conclusions:**

This technique of using the cervix as a natural tamponade appears to be safe, simple, time-saving and potentially effective method for controlling the severe postpartum hemorrhage (PPH) caused by placenta previa/placenta previa accreta. This technique deserves to be one of the tools in the hands of obstetricians who face the life-threatening hemorrhage of placenta accreta.

**Trial registration:**

ClinicalTrials.gov NCT02590484. Registered 28 October 2015

**Electronic supplementary material:**

The online version of this article (doi:10.1186/s12884-015-0731-9) contains supplementary material, which is available to authorized users.

## Background

Patients with placenta previa (PP) may develop severe postpartum hemorrhage after removal of the placenta [1]. Moreover, abnormal adherence of the placenta to the myometrium (placenta accreta) may be associated with life-threatening maternal hemorrhage due to its incomplete separation [2].

Normally, the placenta adheres only to the decidua basalis, thus it separates smoothly from the wall of the uterus after delivery [2]. Placenta accreta (PA) exists when the chorionic villi penetrate through the decidua basalis into the myometrium [3]. Morbidly adherent placenta is a spectrum according to the degree of invasion of the placenta into the uterine wall. The placenta is called accreta when it invades the myometrium superficially. Placenta increta exists when the chorionic villi invade the myometrium more deeply. Placenta percreta involves invasion of the placenta to the uterine serosa, and this might involve other nearby organs such as the urinary bladder; however, the term “accreta” is frequently used for the three types [2, 4, 5].

Currently, there is dramatic increase in the incidence of placenta previa and placenta accreta due to the increasing rate of cesarean delivery combined with increasing maternal age [3, 5].

Placenta previa and placenta accrete carry significant maternal and fetal morbidity and mortality [3]. The maternal mortality in women with PA may reach as high as 7–10 % [3, 6–8].

Several techniques have been described in the literature for controlling massive bleeding associated with placenta previa cesarean sections, including uterine packing with gauze [9], balloon tamponades [10], the B-Lynch suture [11], insertion of parallel vertical compression sutures [1], a square suturing technique [12] and embolization or ligation of the uterine and internal iliac arteries [13], but there is a wide variation in the success rate of these maneuvers [14, 15].

In a case report, Dawlatly et al. [16] described a simple technique of suturing an inverted lip of the cervix over the bleeding placental bed that was successful in controlling the bleeding, saving the patient’s life, and preserving her uterus.

Here, we present our experience with the use of this Dawlatly stitch in 40 cases of placenta previa and/or placenta previa accreta.

## Methods

This prospective study was conducted on 40 pregnant women admitted to our hospital between June 2012 and November 2014. All participating women had one or more previous cesarean deliveries and were diagnosed with placenta previa and/ or placenta previa accreta by ultrasound. When the ultrasound result was not conclusive for placenta accreta, MRI was performed. All women meeting inclusion criteria were approached. However, ten women declined participation in the study.

All participating women desired to preserve their fertility. They were counseled properly and were given clear information about the diagnosis, the risk of severe postpartum hemorrhage and the methods that can be used to control this massive hemorrhage, including conservative methods and radical method (emergency hysterectomy). They were informed that cesarean hysterectomy is the 1^st^ option in case of placenta percreta, diffuse placenta accreta or increta and in the presence of uncontrollable hemorrhage and these were considered as exclusion criteria for conservative management. The conservative methods used were explained to the patients and included the suture technique described in this study, uterine and/or internal iliac artery ligation. All participants signed a written informed consent about the procedure(s), the risks including massive postpartum hemorrhage (PPH) and the need for blood transfusion, the use of conservative methods and the possibility of proceeding to emergency hysterectomy with the accompanying risk of injury to adjacent structures as the bladder, ureters and bowel.

This study was approved by the ethical committee of the Department of Obstetrics and Gynaecology, Minia University Hospital on 17/03/2012 (Registration number: MUH14367). The hospital protocol for the management of placenta previa and placenta accreta was followed. The surgical steps were as follows:

1. General or spinal anesthesia is administered according to the hemodynamic state of the patient, suspicion of PA and the opinion of the anesthetic and surgical teams.
2. The patient was placed in a dorsal lithotomy position with hip abduction and flexion to observe the amount of blood loss and to facilitate vaginal access if needed such as elevating the cervix from the vaginal aspect to facilitate its grasping.
3. Cesarean section with a Pfannensteil or vertical midline incision was performed. Good reflection of the urinary bladder was performed before making the uterine incision.
4. If the placenta separated completely, the placental bed was observed for bleeding and managed accordingly. If the placenta did not separate at all (diffuse placenta accreta), we proceeded to either cesarean hysterectomy or the placenta was left in place. In cases with partial placental separation (focal placenta accreta), the remaining portion of the placenta was removed, and the placental bed was observed to determine the amount of bleeding and the need for intervention. Excessive bleeding from the placental bed which soaks more than three swabs within a short time with blood seen pouring from the placental bed into the lower uterine segment was considered significant blood loss that warrants surgical intervention.
5. If the bleeding originated primarily from the anterior portion of the lower uterine segment, the surgeon introduced his/her hand through the uterine incision into the lower uterine segment until it touched the cervix. A long Allis forceps was passed through the uterine incision and used to grasp the anterior lip of the cervix, pulling the cervix upwards into the uterine cavity. An assistant was sometimes needed to elevate the cervix upwards from the vaginal aspect. The anterior lip of the cervix was then sutured to the anterior wall of the lower uterine segment using two or three simple interrupted absorbable stitches (Vicryl or Vicryl rapide no. 0). This aids to compress the bleeding sites of the placental bed and support the very thin lower uterine segment seen in such cases. If the placenta was implanted posteriorly and the bleeding areas were mainly from the posterior wall of the uterus, the same procedure could be repeated using the posterior lip of the cervix, which could then be sutured to the posterior wall of the lower uterine segment. A Hegar dilator was inserted in a retrograde manner from the abdominal aspect to ensure patency of the cervical canal during the suturing process.
6. The bleeding was then observed from the abdominal aspect and also vaginally.
7. At the end, the uterine incision was closed in the usual way.
8. Postoperatively, the patients were observed in the ward or ICU according to their degree of hemodynamic stability.
9. The patients were then debriefed and given a clear explanation about the events, procedures performed and the expected duration of hospital stay.
10. All patients were given two follow-up appointments 3 and 6 months after delivery. During each appointment, history was taken and speculum examination was performed to observe the morphological picture and the anatomical position of the cervix. Ultrasound examination of the uterus was also performed to exclude the presence of haematometra as a consequence of cervical stenosis.

**Fig. 1:**
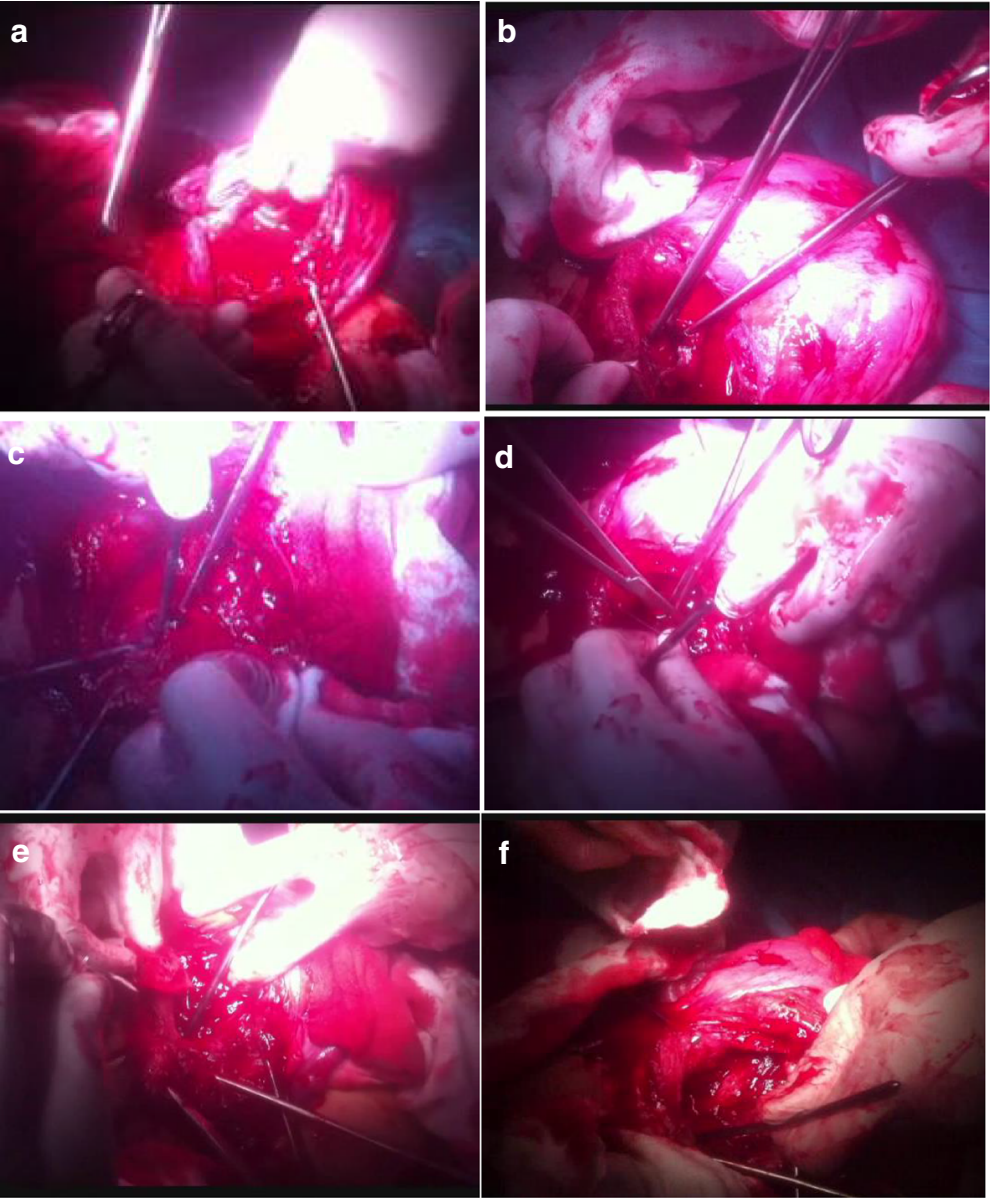
Summary of the steps of the technique of cervical inversion. **a**: The placental bed showing significant bleeding. **b**: The inverted cervical lips grasped by 2 Allis forceps. **c**: The inverted cervical lips with Hegar dilator in between. **d**: Suturing the posterior cervical lip to the posterior wall of the lower uterine segment. **e**: Suturing the anterior cervical lip to the anterior wall of the lower uterine segment. **f**: The end of the procedure with the dilator being removed from the cervical canal

A summary of the above mentioned steps can be seen in Fig. 1. An additional movie file shows this in more detail (see Additional file 1).

### Statistical analysis

Statistical advice regarding study design was obtained from the Statistical Services Unit (SSU) of the University. The data were coded, entered and processed on an IBM-PC compatible computer using SPSS (version 16, IBM, NY, USA).

Descriptive statistics were performed, including description of quantitative variables as the mean, SD and range, and description of qualitative variables as number and percentage. T-test was performed to compare the mean pre-operative hemoglobin and the mean post-operative hemoglobin.

## Results

Tables 1 and 2 show the demographic and clinical data of the studied group. The mean age was 29.2 ± 2.7 years. Regarding parity, ten patients (25 %) were para 1 and 22 patients (55 %) were para 2. Regarding the number of living children, four patients (10 %) have no children and 12 patients (30 %) have just one child and 18 patients (45 %) have two children which is still considered small family size in our communities.

**Table 1:** Demographic data of the studied group

Characteristic	Mean ± SD	Range	Number (%)
Age (years)	29.2±2.7	25–36	
Parity		P1-P4	
P1			10 (25 %)
P2			22 (55 %)
P3			7 (17.5 %)
P4			1 (2.5 %)
No. of living children		0–3	
No children			4 (10 %)
One child			12 (30 %)
Two children			18 (45 %)
Three children			5 (12.5 %)
Four children			1 (2.5 %)
No. of previous cesarean sections		1–4	
One cesarean section			10 (25 %)
Two cesarean sections			22 (55 %)
Three cesarean sections			7 (17.5 %)
Four cesarean section			1 (2.5 %)
Gestational age (weeks)	5.9±1.1	33–37	

*SD* Standard Deviation, *P* Para, *No* Number

**Table 2:** Clinical data of the studied group

Characteristic	Number (%) (*n*=40)	Mean ± SD	*P* value
Diagnosis			
Placenta accreta	29 (72.5 %)		
Placenta previa major anterior	6 (15 %)		
Placenta previa major posterior	5 (12.5 %)		
Inverted cervical lip			
Both lips inverted	25 (62.5 %)		
Anterior lip inverted	10 (25 %)		
Posterior lip inverted	5 (12.5 %)		
Time required to perform the technique of cervical inversion (min)		5.4 ± 0.6 (range:4.3–7.1 min)	
Hysterectomy needed	2 cases (5 %)		
Intra-operative blood loss (mL)		1572.5 ± 390.2	
Pre-operative hemoglobin (gm/dl)		10.8 ± 0.23	
Post-operative hemoglobin (gm/dl)		9.3 ± 0.22	**< .0001
No. of blood units transfused		3.1 ± 0.6	
Post-operative hospital stay (days)		3.5 ± 0.6	
Complications			
Bladder injury	2 (5 %)		
Wound infection	1(2.5 %)		
Postoperative fever	1 (2.5 %)		
Speculum examination 3 months^a^	35 cases (87.5 %)		
Normal cervix	33/35 (94.2 %)		
Displaced cervix	2/35 (5.8 %)		

^a^Percentages worked on less numbers from the overall as 35 cases only attended their three months follow up appointment.

**P value for the difference between the mean pre-operative hemoglobin and the mean post-operative hemoglobin

Regarding anesthesia, 24 patients (60 %) had general anesthesia and 16 patients (40 %) had spinal anesthesia. Intra-operatively, we identified 29 cases of placenta accreta, six cases of placenta previa major anterior and five cases of placenta previa major posterior. To control bleeding from the placental bed, both the anterior and posterior cervical lips were used in 25 cases, the anterior lip only was used in ten cases and the posterior lip in five cases. The mean time needed to perform the technique was 5.4 ± 0.6 min (range: 4.3–7.1 min). The technique of cervical inversion described above was successful in stopping the bleeding in 38 out of 40 patients; yielding a success rate of 95 %. We resorted to hysterectomy in only two cases (5 %). Histopathological examination of the uterus in these two cases showed placenta increta. The mean intra-operative blood loss was 1572.5 mL, and the mean number of blood units transfused was 3.1. The difference between the mean pre-operative hemoglobin (10.8 ± 0.23 gm/dl) and the mean post-operative hemoglobin (9.3 ± 0.22 gm/dl) was statistically significant (*p* value < .0001). The complications encountered were as follows: bladder injury in the two patients who underwent hysterectomy and wound infection in one patient. Postoperative fever that responded to antibiotics occurred in one patient. The mean duration of the postoperative hospital stay was 3.5 days. All women were given two follow-up appointments 3 and 6 months following delivery. At the 3 months follow up appointment, speculum examination was performed in only 35 cases (87.5 %), as the other five cases (12.5 %) did not attend their appointment. Speculum examination revealed normal position and normal morphology of the cervix in 33 cases. In two patients, the cervix was displaced upwards. Colposcopic examination of the cervix in the two patients showed unremarkable findings. In the same sitting, office hysteroscopy was done and the cavity was normal with no evidence of intrauterine synechiae in these two patients. Thirty patients (75 %) attended their 6 months follow-up appointment. Menstruation was resumed in 20 (66.7 %) patients. The other ten patients (33.3 %) were amenorrheic which could be due to lactation or another cause. We are still following up these ten patients for a longer period of time to see if this amenorrhea was lactational or pathological. Ultrasound examination of the uterus and speculum examination of the cervix were unremarkable. All patients were advised to contact the hospital in case of experience of any unusual symptoms or occurrence of pregnancy.

## Discussion

The management options in PPH associated with placenta previa and placenta accreta include either (1) a radical approach (surgical removal of the uterus and the involved tissues e.g., partial cystectomy if the bladder is involved) or (2) a conservative approach [4, 17]. Although hysterectomy is still the recommended treatment for placenta accreta [17–19], when placenta percreta involves adjacent structures, the bleeding might be severe, and surgery is very risky, with the possibility of damage to these structures due to the morbidly adherent placenta [4, 20]. clearly, this radical approach is unacceptable to women with low parity who desire uterine preservation [3]. In some cultures, many people consider women who have had a hysterectomy as having lost the most important aspect of their feminine character. This could have a major impact on the psychological state of these women and their quality of life.

Many conservative management options have been advocated in the literature [4]. The 1st option is leaving the placenta completely or partially in place with or without administering methotrexate [21]. Although this approach of leaving the placenta seems to be a safe option, it is associated with serious complications including either primary or reactionary catastrophic bleeding [22] and infection resistant to antimicrobial therapy, resulting in eventual delayed hysterectomy for some patients [5, 23]. In addition, this approach cannot be used in hemodynamically unstable patients. Added to this, there is controversy regarding management of the placenta left in place. Conservative treatment requires a prolonged period of postpartum follow-up and patient compliance and adherence to treatment as well as consideration of the risk for severe morbidity and possibly mortality for weeks or even months after delivery [7, 20].

Sometimes, the placenta separates partially (focal placenta accreta) or is injured during the delivery of the baby, and it becomes necessary to remove the placenta and control the massive bleeding from the placental bed [3].

In this case series, we present a suture technique which was 1st described by Dawlatly in a case report in 2007.

Grasping the cervical lip(s) and suturing it into the paper-thin lower uterine segment seen in such cases can help to control the massive bleeding and create a good flap that can be used in closing the uterine incision. With this technique, the cervix can be used as a natural tamponade replacing the artificial tamponades that are frequently used for stopping PPH in cases of placenta previa and placenta previa accreta. The cervical canal remains patent, and an absorbable suture material is used to help return the cervix to its original position. There is no risk of injury to the ureters or uterine vessels as the stitches are inserted in the substance of the cervix and the lower uterine segment. When compared to artificial tamponades like Bakri balloon, cervical inversion is a natural method, affordable with no cost and in the same time appears to be safe, potentially effective and more useful in controlling bleeding from the placental bed in the lower uterine segment. On the other side, the long term implications of cervical inversion are still unclear. In some cases (not reported in this case series), we have used other conservative methods like Bakri balloon, uterine and internal iliac artery ligation or combination of these methods. We have found that these methods are more effective than cervical inversion in patients with excessive bleeding from both the upper uterine and lower uterine segments (combined atonic PPH and placental site bleeding). In this case series, we are reporting only the cohort of patients in whom we have used cervical inversion as a single technique to control bleeding from the placental bed.

Our patients were of young age and low parity with a small number of living children. This reflects the importance of using conservative technique(s) to preserve the uterus and fertility of women in the studied group. This technique was introduced in our department and is now widely adopted by our junior and senior staff. In addition, it can be used in cases of repeated cesarean deliveries with very thin lower uterine segment to help close the uterine incision.

Our study is supported by a recent study done by Sakhavar et al. who reported that cervical inversion exerts pressure on the lower segment arteries thus reducing the vascular blood flow leading to relative hemostasis. Sakhavar et al. actually describe a slightly different technique. The cervix is inverted in a similar way to ours, after which the placental bed is sutured to control bleeding. After bleeding is controlled, the cervix is returned to its original position. In their study, cervical inversion was successfully applied to ten cases. In all ten cases, the bleeding was stopped within 3–5 min from the beginning of procedure. They did not report any major complications, and blood transfusions or obstetric hysterectomies were not necessary [24].

The first limitation in interpreting the results of our study is that it is not a randomized trial. However, the case series was prospectively collected, and placement of this suture was associated with good results. Follow-up clinical assessments were also done. So this technique might be effective and could gain widespread acceptance. The other limitation in our study is the uncertainty of the effect of this technique on the anatomical and functional capacity of the cervix and its impact on future pregnancy and delivery. However, these worries could be minimized by the promising results of the short and middle term follow-up of these patients.

## Conclusions

Based on these preliminary data of this study, we conclude that this technique of using the cervix as a natural tamponade is apparently safe, simple, time-saving and potentially effective in controlling the severe PPH associated with cases of placenta previa and/or placenta previa accreta. This technique deserves to be one of the tools in the hands of obstetricians who face the life-threatening hemorrhage of cases of placenta accreta. Further studies are needed with extended follow-up of the patients to explore the long-term implications of this technique.

## Additional file

Additional file 1:
**Cervical Inversion_placenta accreta.** This is a movie file showing detailed steps of the technique of cervical inversion. (XML 7 kb)

## Abbreviations

PP: Placenta previa
PA: Placenta accreta
PPH: Postpartum hemorrhage
No.: Number
ICU: Intensive care unit
SPSS: Statistical package for social science
SD: Standard deviation
